# When hosts gather: how extreme seasonal aggregation affects epidemiological outcomes

**DOI:** 10.1016/j.idm.2026.04.004

**Published:** 2026-04-09

**Authors:** Daniel N.R. Longmuir, Simon Johnstone-Robertson, Andrew J. Hoskins, Roslyn I. Hickson, Stephen A. Davis

**Affiliations:** a Mathematical Sciences, STEM College, RMIT University, Melbourne, Australia; b Commonwealth Scientific and Industrial Research Organisation (CSIRO), Townsville, Australia; c Australian Institute of Tropical Health and Medicine, James Cook University, Townsville, Australia; d North Australian Indigenous Land and Sea Management Alliance (NAILSMA), Darwin, Australia; e School of the Environment, University of Queensland, Brisbane, Australia

**Keywords:** Mathematical modelling, Metapopulation, SIR model, Seasonal aggregation, Wildlife disease

## Abstract

Wildlife aggregate for many reasons (e.g. reproduction, feeding) and at times these aggregations can be extreme, with host densities increasing several orders of magnitude. While the impact of seasonality on infectious disease dynamics is well studied, few—if any—studies have explicitly examined how extreme aggregation affects key epidemiological outcomes. Here we consider an epidemic in a closed SIR (Susceptible–Infectious–Recovered) metapopulation with a hub–satellite structure, where seasonal movement into the hub follows a modified Gaussian function. We numerically explore how aggregation duration and timing shape two outcomes: final size and peak prevalence. We find a narrow set of circumstances and pathogens for which even extreme aggregation materially alters these outcomes. When aggregation coincides with, or begins just prior to, infection introduction, aggregation can strongly affect pathogens with $R_{0}\approx 1$ or $R_{0}<1$, enabling epidemics that would otherwise fade. Effects are strongest under density-dependent transmission, where contact rate scales with local density; frequency-dependent transmission renders aggregation negligible. High transmissibility $\left(R_{0}\gg 2\right)$ minimises aggregation's impact because most susceptibles are infected regardless of density changes.

## 1 Introduction

A common ecological phenomenon is the regular aggregation of individuals of the same (or sometimes multiple) species at particular times to satisfy life history requirements. For example animals moving to a specific area to reproduce or access seasonal resources (Loiselle & Blake, 1991; Phipps et al., 2019; Rushing et al., 2017). These episodic, and sometimes massive, increases in local population density can profoundly affect ecological and epidemiological dynamics of the whole metapopulation through increased contact rates and pressures on local resources (Becker et al., 2020; Lloyd & May, 1996). Understanding how these periodic increases in local density influence disease dynamics is essential for improving theoretical and applied frameworks in disease ecology (Lunn et al., 2021).

Epidemiological models are valuable tools for investigating how aggregation events influence pathogen dynamics (Han et al., 2021). The foundational susceptible–infected–recovered (susceptible–infectious–recovered (SIR)) model introduced by Kermack and McKendrick (Kermack et al., 1927, 1932, 1933) remains central to understanding the spread and persistence of infectious disease. By compartmentalising populations based on their disease status, these models have demonstrated how transmission and recovery rates shape epidemic trajectories. While originally applied to well-mixed populations, the adaptability of SIR-based analysis has enabled extensions to explore the spatial and temporal complexities of wildlife systems (Brown & Gilligan, 2014; Jeong & McCallum, 2021; Lloyd-Smith et al., 2005).

One key extension involves metapopulation models, which divide populations into discrete, homogeneous patches connected by migration (Hanski, 1999; Levins, 1969). These models are well-suited for studying wildlife diseases in populations that exhibit seasonal movement and aggregation (Jeong & McCallum, 2021; Plowright et al., 2011), since they allow one to explore how migration rates and patch connectivity affect disease dynamics. Many metapopulation epidemic models assume frequency-dependent transmission within patches when local populations are treated as internally homogeneous and coupled through migration (Dhirasakdanon & Sattenspiel, 2007; Hess, 1996). Likewise, seasonality is often introduced through time-varying transmission or host demography, such as seasonal birth pulses (Grassly & Fraser, 2006; Peel et al., 2014). In wildlife systems, however, seasonal aggregation can also alter local host density substantially, making density-dependent contact processes biologically relevant (Berg et al., 2018; McCallum et al., 2001).

The link between host aggregation and pathogen dynamics can be seen across diverse wildlife systems. Seasonal migrations of waterfowl, for example, create vast networks connecting geographically distant populations, facilitating the continental and intercontinental spread of Avian Influenza Viruses (Avian Influenza Virus (AIV)) as birds congregate at stopover and wintering sites (Olsen et al., 2006). Similarly, freshwater fish like Common Carp (*Cyprinus carpio*) exhibit intense, localised aggregation during spawning seasons, creating conditions conducive to the rapid transmission of pathogens such as Cyprinid Herpesvirus 3 (Cyprinid Herpesvirus 3 (CyHV-3)) within dense breeding groups (Durr et al., 2019; Uchii et al., 2010). Furthermore, highly mobile species like *Pteropid* bats (flying foxes) form large roosts that can serve as reservoirs for zoonotic pathogens like Hendra virus (Hendra Virus (HeV)), with their movement patterns influencing both viral persistence within bat populations and the potential for spillover to other species (Plowright et al., 2015).

This study extends a classical SIR metapopulation model to incorporate seasonally recurrent aggregation events to explore how these events affect the outcomes of an epidemic within a fully susceptible, closed population. Seasonal movement is modelled as a modified Gaussian function, which captures the temporal dynamics of aggregation and subsequent dispersal. In contrast to approaches that impose seasonality directly through transmission or recruitment, the novelty of the present framework lies in representing seasonality through background host movement into and out of a centralised node. This work provides a simple framework which links spatial structure, temporal variability, and pathogen transmission in wildlife which display these aggregation behaviours.

## 2 Material and methods

### 2.1 Population model with aggregation function

We develop a metapopulation model to investigate how seasonal aggregation events influence epidemic final size in a closed, fully susceptible population. Although motivated by highly mobile taxa that exhibit large-scale aggregations and migrations—e.g. flying foxes (Pteropus spp.), waterfowl and common carp (*Cyprinus carpio*)—the model aims to build foundational understanding of aggregation effects rather than examine any one taxon in detail.

**Fig. 1:**
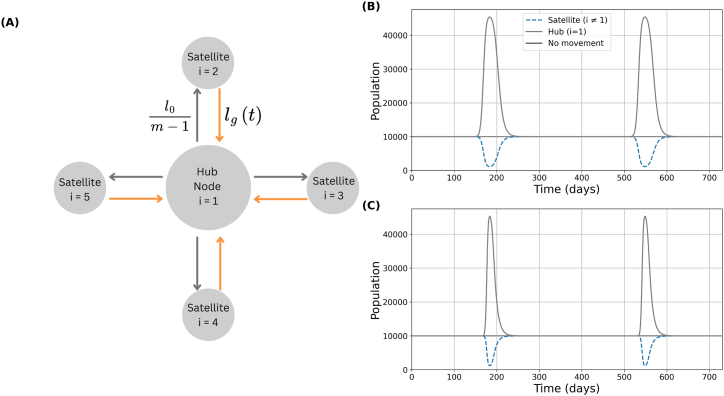
**Metapopulation structure and movement dynamics.** (**A**) Hub-satellite network structure (*m* = 5) with baseline movement out of the hub at rate *l*_0_/(*m* − 1) (grey arrows) and seasonal movement into the hub at rate *l*_*g*_(*t*) (orange arrows). (**B**) Population trajectories for *b* = 10. (**C**) Population trajectories for *b* = 20. In Panels B and C, the solid grey line shows the hub population *N*_1_(*t*), the blue dashed line shows the population of a representative satellite node *N*_*i*_(*t*) (*i* ≠ 1; all satellites are identical by symmetry), and the black line shows the population dynamics with no aggregation present. Initial conditions are *N*_1_(0) = *N*_*i*_(0) = 10^4^ for all patches.

The system consists of a central hub (*i* = 1) and *m* − 1 satellite nodes (*i* ≠ 1), for a total of *m* nodes. This mainland-island (Hanski & Gilpin, 1991), or hub-and-spoke, metapopulation structure is chosen as a representation of many ecological aggregation scenarios, such as breeding grounds or key resource location (hub) drawing individuals temporarily from surrounding territories (satellites), allowing for a focused analysis of density changes within the primary aggregation site (Fulford et al., 2002). Population movement between nodes is governed by a time-dependent aggregation rate (*l*_*g*_(*t*)), representing the seasonally recurrent influx of individuals into the central hub, and a constant baseline migration rate (*l*_0_) representing movement out of the hub. This asymmetry—variable influx *l*_*g*_(*t*) and constant outflow *l*_0_—models how biological cues (e.g. breeding seasons or resource pulses) drive individuals toward the hub. Dispersal back to satellites, by contrast, is treated as a constant background process. As shown in Fig. 1A, the hub receives individuals from each satellite at rate *l*_*g*_(*t*), while each satellite receives individuals from the hub at rate *l*_0_/(*m* − 1). The total population size remains constant, reflecting a closed system with no natural births or deaths. The equations governing the population movement dynamics are

(1) $$\begin{array}{ll} \frac{dN_{1}}{dt} & =\overset{m}{\underset{i=2}{\sum }}l_{g}\left(t\right)N_{i}-l_{0}N_{1},\\ \frac{dN_{i}}{dt} & =\frac{l_{0}N_{1}}{m-1}-l_{g}\left(t\right)N_{i},\;\text{for }i=2,\ldots ,m \end{array}$$

The movement rate into the hub *l*_*g*_(*t*) is modelled as a periodic Gaussian pulse,

(2) $$l_{g}\left(t\right)=\frac{l_{0}}{m-1}+\left(l_{\max}-\frac{l_{0}}{m-1}\right)e^{-{\left(b\cos\left(\frac{\pi }{365}t\right)\right)}^{2}}.$$

Formulations of this kind have previously been used to model seasonality in demographic processes, particularly seasonal birth pulses (Peel et al., 2014). Here, we adapt that general idea to describe seasonal movement into the hub. Equation (2) was chosen so that movement into the hub varies seasonally around a baseline state *l*_0_/(*m* − 1) in which all movement rates into the hub are equal when aggregation is not occurring. The sinusoidal term adds a bounded seasonal pulse, with annual periodicity generated by the cos^2^(*πt*/365) expression. The parameter *b* controls the width of this pulse: larger values produce shorter, sharper aggregation events, whereas smaller values produce broader aggregation periods, while *l*_max_ sets the peak migration intensity.

Fig. 1B and C illustrate the population dynamics for the populations in the hub (*i* = 1) and satellite nodes (*i* ≠ 1) resulting from different aggregation durations, controlled by the aggregation parameter *b*. These specific values, *b* = 10 and *b* = 20, were chosen to clearly demonstrate the effect of this parameter: Fig. 1B shows the dynamics for *b* = 10, corresponding to a longer aggregation pulse lasting approximately 60 days, while Fig. 1C shows the dynamics for *b* = 20, resulting in a shorter aggregation period of approximately 30 days. In both panels, the solid grey line shows the hub population *N*_1_(*t*), while the blue dashed line shows a representative satellite population *N*_*i*_(*t*), with all satellite nodes behaving identically by symmetry. In both scenarios, the hub population increases during each aggregation pulse due to the higher inward flow rate *l*_*g*_(*t*), while satellite populations decrease correspondingly. The black line shows the population dynamics with no aggregation present, and the initial conditions are *N*_1_(0) = *N*_*i*_(0) = 10^4^ for all patches. No natural births or deaths occur, maintaining a fixed total population size across the metapopulation.

### 2.2 SIR metapopulation model

To simulate disease spread, we couple the metapopulation model (Equations (1), (2)) with an SIR model where the population in each node *i*(*i* = 1, *…*, *m*) is compartmentalised into those susceptible (*S*_*i*_), infectious (*I*_*i*_) or recovered (*R*_*i*_).The total population of each patch *i* is

(3) $$N_{i}=S_{i}+I_{i}+R_{i}.$$

We define a generalised contact-rate function

(4) $$c_{i}=\kappa \;{\left(\frac{N_{i}}{A}\right)}^{1/q},$$

which is used here as a simple scaling law spanning the classical density-dependent and frequency-dependent transmission limits (Durr et al., 2019; Smith et al., 2009). When *q* = 1, contact rate scales linearly with density, corresponding to density-dependent transmission. In the limit *q* → *∞*, contact rate becomes independent of density, corresponding to frequency-dependent transmission. Intermediate values 1 < *q* < *∞* represent a saturating dependence of contact on density. The corresponding transmission rate is

(5) $$\beta =\frac{\kappa \;v}{A^{1/q}},$$

where *v* represents the per-contact transmission probability, and *A* is the fixed area of each node. Together, these yield the site-specific force of infection

(6) $$\lambda_{i}=\beta \;I_{i}\;N_{i}^{\frac{1}{q}-1}.$$

Combining movement and transmission yields the full SIR system. For the hub:

(7) $$\begin{array}{ll} \frac{dS_{1}}{dt} & =-\beta \;S_{1}\;I_{1}\;N_{1}^{\frac{1}{q}-1}+\overset{m}{\underset{i=2}{\sum }}l_{g}\left(t\right)\;S_{i}-l_{0}\;S_{1},\\ \frac{dI_{1}}{dt} & =\beta \;S_{1}\;I_{1}\;N_{1}^{\frac{1}{q}-1}-\gamma \;I_{1}+\overset{m}{\underset{i=2}{\sum }}l_{g}\left(t\right)\;I_{i}-l_{0}\;I_{1},\\ \frac{dR_{1}}{dt} & =\gamma \;I_{1}+\overset{m}{\underset{i=2}{\sum }}l_{g}\left(t\right)\;R_{i}-l_{0}\;R_{1}, \end{array}$$

and for each satellite:

(8) $$\begin{array}{ll} \frac{dS_{i}}{dt} & =-\beta \;S_{i}\;I_{i}\;N_{i}^{\frac{1}{q}-1}+\frac{l_{0}\;S_{1}}{m-1}-l_{g}\left(t\right)\;S_{i},\\ \frac{dI_{i}}{dt} & =\beta \;S_{i}\;I_{i}\;N_{i}^{\frac{1}{q}-1}-\gamma \;I_{i}+\frac{l_{0}\;I_{1}}{m-1}-l_{g}\left(t\right)\;I_{i},\\ \frac{dR_{i}}{dt} & =\gamma \;I_{i}+\frac{l_{0}\;R_{1}}{m-1}-l_{g}\left(t\right)\;R_{i}. \end{array}$$

Table 1 summarises the parameter values selected to broadly reflect the ecology of highly mobile, aggregating wildlife and their pathogens. Movement dynamics were informed by telemetry studies of Little Red Flying Foxes (Westcott et al., 2020; Welbergen et al., 2020), motivating a peak aggregation rate *l*_max_ = 1 day^−1^ to capture rapid nightly decampment toward a central hub during seasonal influxes (Roberts et al., 2012), and a baseline migration rate *l*_0_ = 0.1 day^−1^ to represent significant background mobility (Roberts et al., 2012). We explore two aggregation pulse widths, *b* = 10 and *b* = 20, corresponding to approximately 60-day and 30-day events, respectively, consistent with typical breeding or migratory periods across taxa. We examined three introduction times, *t*_event_ ∈ {90, 160, 170} days, to assess how epidemic seeding before and near aggregation pulses influences outbreak trajectories. An initial population size of *N*_*i*_ = 10^4^ per node reflects observed LRFF roost sizes (Plowright et al., 2011) and provides sufficient scale to examine density-driven epidemic dynamics.

**Table 1:** Key model parameters and baseline values.

Parameter	Description	Value	Units	Sources
*l*_0_	Baseline migration rate	0.1	day^−1^	(Roberts et al., 2012)
*l*_max_	Peak aggregation rate	1	day^−1^	(Westcott et al., 2020; Welbergen et al., 2020; Roberts et al., 2012)
*b*	Aggregation pulse width	{10, 20}	–	Assumed
*t*_event_	Time of infection introduction	{90, 160, 170}	day	Assumed
*q*	Transmission scaling exponent	{1, 2, 5, *∞*}	–	Assumed
*γ*	Recovery rate	[0.1, 0.5]	day^−1^	(Plowright et al., 2011; Jeong and McCallum, 2021; Roche et al., 2009; Saenz et al., 2012; Breban et al., 2009; Tolo et al., 2021)
*β*	Transmission rate (*q* = 1)	[10^−5^, 10^−4^]	capita^−1^ day^−1^	Literature-based $R_{0}$ (Wang et al., 2013; IGLESIAS et al., 2010; Grear et al., 2017; Epstein et al., 2020)

The epidemiological parameters were chosen to span biologically plausible ranges and facilitate exploration across a broad spectrum of baseline transmission scenarios. Recovery rates (*γ*) of 0.1 to 0.5days^−1^ correspond to infectious periods of 2 to 10 days as reported for Hendra virus, avian influenza virus, and cyprinid herpesvirus 3 (Breban et al., 2009; Jeong & McCallum, 2021; Plowright et al., 2011; Roche et al., 2009; Saenz et al., 2012; Tolo et al., 2021). In the case *q* = 1, the density dependent transmission rate (*β*) varies from 10^−5^ to 10^−4^ and produces basic reproduction numbers $\left(R_{0}\right)$ that range from 0.2 to 10 for initial population sizes of *N*_1_(0) = *N*_*i*_(0) = 10^4^, spanning published and model-implied $R_{0}$ values for avian influenza viruses in wild birds, and Hendra and Nipah viruses in flying foxes (Epstein et al., 2020; Grear et al., 2017; IGLESIAS et al., 2010; Wang et al., 2013).

### 2.3 Epidemiological outcomes

Exploration of the SIR metapopulation model (Equations (7), (8))) was undertaken to quantify epidemic outcomes under both extreme seasonal aggregation and “no aggregation” scenarios. The chosen epidemiological outcomes were the total number in the recovered state (final size) and the maximum number infectious across all nodes at any given time (peak prevalence). A “no aggregation” scenario corresponds to *l*_*g*_(*t*) = *l*_0_/(*m* − 1) *∀ t* such that while there is still movement between patches, there are no changes in population density in any patch. For each combination of transmission rate *β*, recovery rate *γ*, aggregation width *b*, and introduction time *t*_event_, we computed the final size (FS) and peak prevalence (PP) as:

(9) $$FS=\overset{m}{\underset{i=1}{\sum }}R_{i}\left(end\right),$$

and

(10) $$PP=\max_{t}\overset{m}{\underset{i=1}{\sum }}I_{i}\left(t\right).$$

Seasonal effects were measured by

(11) $$\Delta FS={FS}_{\left(agg\right)}-{FS}_{\left(base\right)},$$

and

(12) $$\Delta PP={PP}_{\left(agg\right)}-{PP}_{\left(base\right)},$$

the differences between aggregation and no aggregation outcomes.

### 2.4 Numerical simulation

Simulations were conducted in Python 3.10 (Van Rossum & Drake, 2009) using SciPy's solve_ivp interface (Virtanen et al., 2020) with the DOP853 solver. Integration tolerances were set such that total population size $N=\sum_{i=1}^{m}N_{i}\left(t\right)$ remained conserved to within 0.01% across the simulation period. The system of differential equations (Equations (7), (8))) was integrated over a 5-year horizon (1825 days).

Transmission and recovery rates were taken from the ranges in Table 1 using 100 evenly spaced values for each parameter. Epidemics were initiated by introducing a single infected individual into the hub node at *t*_event_ ∈ {90, 160, 170} days. The latter two timings correspond to introductions on or near the onset of the aggregation (*t*_event_ = 160 for *b* = 10 and *t*_event_ = 170 for *b* = 20).

Termination was defined as either 365 consecutive days with no new infections or computational fade-out, where $\sum_{i=1}^{m}I_{i}\left(t\right)<1$ resulted in all remaining infectious individuals being reassigned to the recovered class. All simulations terminated from one of these conditions within the defined period.

## 3 Results

### 3.1 Impact of aggregation on $R_{0}$ (t)

Seasonal aggregation changes patch population sizes over time, and therefore contact opportunities and invasion potential, also vary with calendar time. We define a time-dependent basic reproduction number, $R_{0}\left(t\right)$, as the expected number of secondary infections generated by a typical infectious individual introduced at time *t* into an otherwise fully susceptible metapopulation. We derive this $R_{0}\left(t\right)$ via the next generation matrix (NGM) method (Diekmann et al., 1990, 2010) using epidemiological reasoning, and we confirm this expression using the methods outlined in (Diekmann et al., 2010) (working not shown). We define two types-at-infection that capture the metapopulation structure of the host population, namely those that were infected in the hub (host type 1) and those that were infected in a satellite patch (host type 2). The corresponding next generation matrix is

(13) $$K=\left(\begin{array}{ll} k_{11} & k_{12}\\ k_{21} & k_{22} \end{array}\right),$$

where *k*_*ij*_ represents the expected number of secondary infections in host type *i* caused by a single infectious individual of host type *j*. All elements of the next generation matrix are non-zero since any host type can infect any other host type due to movement between the hub and satellite patches.

We next derive *k*_11_, the expected number infected in the hub by a single infectious host, who itself was infected in the hub. It can be mathematically expressed as the product of the contact rate (Equation (4)), the per-contact probability of infection (*v*), the average infectious period (1/*γ*) and the proportion of time spent in the hub (*ρ*_11_),

(14) $$k_{11}=\beta \;N_{h}^{\frac{1}{q}}\cdot \frac{1}{\gamma }\cdot \rho_{11}$$

where *N*_*h*_ is the total number of individuals in the hub and *N*_*s*_ is the population size of any given satellite patch. On average, an infectious individual in the hub either recovers at rate *γ* or moves out at rate *l*_0_. The probability of recovering before leaving is

(15) $$Pr\left(\text{recovery}\right)=\frac{\gamma }{\gamma +l_{0}}$$

and the probability of a typical individual leaving before recovering is

(16) $$Pr\left(\text{leave}\right)=\frac{l_{0}}{\gamma +l_{0}}.$$

Once a hub-infected individual is in a satellite the probability, on average, of returning to the hub before recovering is

(17) $$Pr\left(\text{return}\right)=\frac{l_{g}\left(t\right)}{\gamma +l_{g}\left(t\right)}.$$

Accounting for movement back and forth between the hub and any satellite patch whilst infectious, the fraction of time spent in the hub can be expressed as a geometric series:

(18) $$\rho_{11}=\frac{\gamma }{\gamma +l_{0}}\overset{\infty }{\underset{n=0}{\sum }}{\left(\frac{l_{0}}{\gamma +l_{0}}\frac{l_{g}\left(t\right)}{\gamma +l_{g}\left(t\right)}\right)}^{n},$$

which converges to

(19) $$\rho_{11}=\frac{\gamma +l_{g}\left(t\right)}{\gamma +l_{0}+l_{g}\left(t\right)}.$$

Substituting Equation (19) into Equation (14) gives

(20) $$k_{11}=\frac{\beta \;N_{1}^{\frac{1}{q}}}{\gamma }\frac{\gamma +l_{g}\left(t\right)}{\gamma +l_{0}+l_{g}\left(t\right)}.$$

Repeating this process for all types-at-infection produces the following expressions for the remaining next generation matrix elements (Equation (13)):

(21) $$\begin{array}{ll} k_{12} & =\frac{\beta \;N_{1}^{\frac{1}{q}}}{\gamma }\frac{l_{g}\left(t\right)}{\gamma +l_{0}+l_{g}\left(t\right)},\\ k_{21} & =\frac{\beta \;N_{i}^{\frac{1}{q}}}{\gamma }\frac{l_{0}}{\gamma +l_{0}+l_{g}\left(t\right)},\\ k_{22} & =\frac{\beta \;N_{i}^{\frac{1}{q}}}{\gamma }\frac{\gamma +l_{0}}{\gamma +l_{0}+l_{g}\left(t\right)}. \end{array}$$

Substituting Equations (20), (21) into (13) yields the full NGM:

(22) $$K=\left(\begin{array}{ll} \frac{\beta N_{1}^{\frac{1}{q}}}{\gamma }\frac{\gamma +l_{g}\left(t\right)}{\gamma +l_{0}+l_{g}\left(t\right)} & \frac{\beta N_{1}^{\frac{1}{q}}}{\gamma }\frac{l_{g}\left(t\right)}{\gamma +l_{0}+l_{g}\left(t\right)}\\ \frac{\beta N_{i}^{\frac{1}{q}}}{\gamma }\frac{l_{0}}{\gamma +l_{0}+l_{g}\left(t\right)} & \frac{\beta N_{i}^{\frac{1}{q}}}{\gamma }\frac{\gamma +l_{0}}{\gamma +l_{0}+l_{g}\left(t\right)} \end{array}\right)=\frac{1}{\gamma \left(\gamma +l_{0}+l_{g}\left(t\right)\right)}\left(\begin{array}{lll} \beta N_{1}^{\frac{1}{q}}\left(\gamma +l_{g}\left(t\right)\right) & \beta N_{1}^{\frac{1}{q}}l_{g}\left(t\right) & \\ \beta N_{i}^{\frac{1}{q}}l_{0} & \beta N_{i}^{\frac{1}{q}}\left(\gamma +l_{0}\right) \end{array}\right)$$

By taking the spectral radius of the next-generation matrix (Equation (22)), we arrive at the time dependent basic reproduction number $R_{0}\left(t\right)$

(23) $$R_{0}\left(t\right)=\frac{1}{2\;\gamma \;\left(\gamma +l_{0}+l_{g}\left(t\right)\right)}\left[\beta \left(N_{1}^{\frac{1}{q}}\left(\gamma +l_{g}\left(t\right)\right)+N_{i}^{\frac{1}{q}}\left(\gamma +l_{0}\right)\right)+\sqrt{\Delta }\right],$$

with

(24) $$\Delta ={\left[\beta \left(N_{1}^{\frac{1}{q}}\left(\gamma +l_{g}\left(t\right)\right)-N_{i}^{\frac{1}{q}}\left(\gamma +l_{0}\right)\right)\right]}^{2}+4\beta^{2}\;N_{1}^{\frac{1}{q}}\;N_{i}^{\frac{1}{q}}\;l_{0}\;l_{g}\left(t\right).$$

In the case where movement rates balance and cause population sizes in every patch to be identical (*N*_1_ = *N*_*i*_ = *N ∀ t*), such that no aggregation occurs, Equation (23) simplifies to

(25) $$R_{0}=\frac{\beta \;N^{\frac{1}{q}}}{\gamma },$$

and so we recover the classical basic reproduction number. To derive this simplified expression, in Equation (23), set *N*_1_ = *N*_*i*_ = *N*. Then

(26) $$\Delta ={\left[\beta N^{1/q}\left(\left(\gamma +l_{g}\left(t\right)\right)-\left(\gamma +l_{0}\right)\right)\right]}^{2}+4\beta^{2}N^{2/q}l_{0}l_{g}\left(t\right)$$

(27) $$=\beta^{2}N^{2/q}{\left(l_{g}\left(t\right)+l_{0}\right)}^{2},$$

so that

(28) $$\sqrt{\Delta }=\beta N^{1/q}\left(l_{g}\left(t\right)+l_{0}\right).$$

and simplifying gives

(29) $$R_{0}\left(t\right)=\frac{\beta N^{1/q}\left[\left(\gamma +l_{g}\left(t\right)\right)+\left(\gamma +l_{0}\right)+\left(l_{g}\left(t\right)+l_{0}\right)\right]}{2\gamma \left(\gamma +l_{0}+l_{g}\left(t\right)\right)}$$

(30) $$=\frac{\beta N^{1/q}}{\gamma },$$

recovering the classical expression.

### 3.2 Impact of aggregation on the epidemiological outcomes

We next explore the broad impacts that aggregation can have on an epidemic in a closed population. We first illustrate the impact by selecting a sample of timeseries to show how an extreme aggregation event can extend or even rescue an epidemic. Secondly, we demonstrate that aggregation has the greatest effect on an epidemic when transmission is density dependent (*q* = 1). Finally, we investigate a broad range of parameter space to understand in detail the effect aggregation has on final size and peak prevalence.

#### 3.2.1 Time-series of infection and recovery

**Fig. 2:**
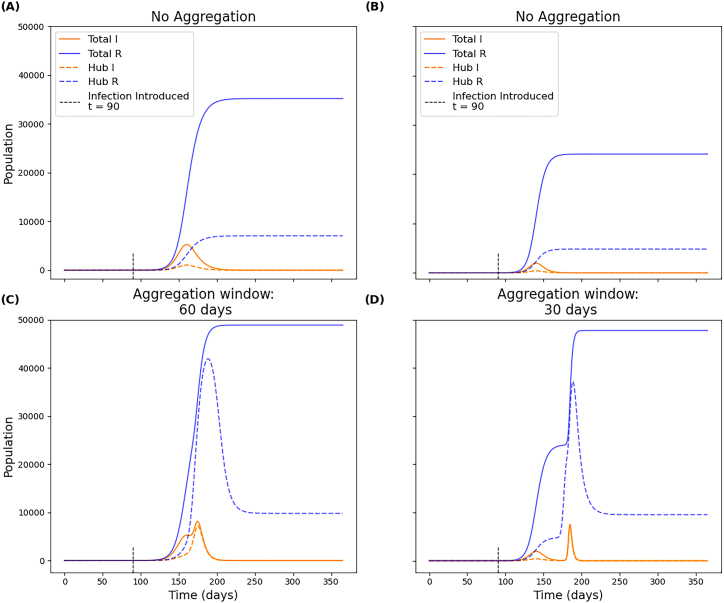
Time-series of infectious (*I*) and recovered (*R*) population trajectories in a hub-and-satellite epidemiological model, with the aggregation parameter *b* controlling the *width* (or duration) of aggregation events under density dependence *q* = 1. Panels (A) and (B) show no aggregation (*l*_*g*_(*t*) = *l*_0_) under two transmission-recovery regimes: *β* = 3.5 × 10^−5^ day^−1^, *γ* = 0.2 day^−1^ (Panel A) and *β* = 6.8 × 10^−5^ day^−1^, *γ* = 0.5 day^−1^ (Panel B). Panels (C) and (D) introduce seasonal aggregation with *b* = 10 (60-day window; *β* = 3.5 × 10^−5^ day^−1^, *γ* = 0.2 day^−1^) and *b* = 20 (30-day window; *β* = 6.8 × 10^−5^ day^−1^, *γ* = 0.5 day^−1^), respectively. Solid lines trace total population counts, while dashed lines trace the hub population. The vertical dashed line marks the time of infection introduction at *t*_event_ = 90 days.

In Fig. 2, the number of infectious and recovered individuals are plotted as a function of time in the absence (Panels A and B) and presence (Panels C and D) of aggregation and for different parameter combinations: *β* = 3.5 × 10^−5^ and *γ* = 0.2 (Panels A and C), and *β* = 6.8 × 10^−5^ and *γ* = 0.5 (Panels B and D). Panel A shows the single-peak epidemic without aggregation, while the longer aggregation (60 days) in Panel C boosts the ongoing outbreak and delays epidemic fadeout due to an extended surge of infectious individuals. Panel B illustrates a single-peak epidemic under no aggregation, and Panel D shows how aggregation can produce a sharp secondary wave, even when the aggregation period is relatively short (30 days). In the absence of aggregation and just prior to its onset, infectious individuals are distributed evenly among the *m* patches; when aggregation occurs, however, the majority of infectious hosts concentrate in the hub.

#### 3.2.2 Effect of transmission-scaling exponent on the final size

**Fig. 3:**
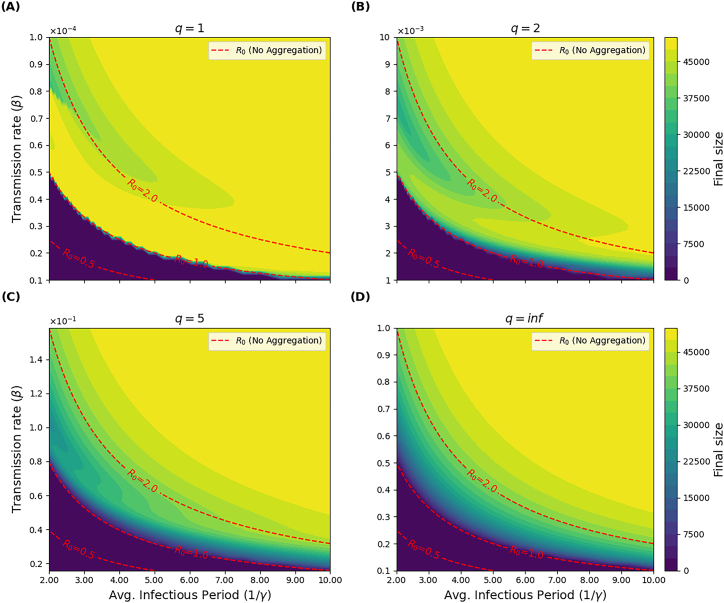
Final size as a function of transmission rate (*β*), average infectious period (1/*γ*) and *q*, for aggregation parameter *b* = 10 with infection introduced at *t*_event_ = 90 days. Panels show four transmission-scaling exponents: *q* = 1 (A), *q* = 2 (B), *q* = 5 (C) and *q* → *∞* (D). Red dashed contours represent the baseline basic reproduction number *R*_0_ = *βN*/*γ* = 0.5, 1.0, 2.0 in the absence of aggregation. As *q* increases, the threshold for large outbreaks moves to higher *β* or longer 1/*γ*, and the intermediate zone narrows. In the *q* → *∞* case, the heatmap perfectly matches what would be observed if there was no aggregation, further emphasising that under frequency dependent transmission, aggregation has no impact.

Fig. 3 shows how the final size varies with transmission rate (*β*) and the average infectious period (1/*γ*) for *q* = 1, 2, 5, *∞*. Under density-dependent transmission (*q* = 1, Panel A), extreme aggregation amplifies the size of outbreaks, especially for $1\leq R_{0}\leq 2$. As *q* increases to 2 and 5 (Panels B−C respectively), transmission depends less on density, so that only larger transmission rates *β* or longer average infectious periods (1/*γ*) yield substantial epidemic peaks. In the frequency-dependent limit (*q* → *∞*, Panel D), where contact rates do not depend on density, aggregation becomes irrelevant. As such, we focus on density dependent transmission (*q* = 1) for the rest of the paper.

#### 3.2.3 Final size and peak prevalence for density dependence

For each combination of *β* and *γ*, two aggregation regimes were considered: *b* = 10 and *b* = 20, corresponding to aggregation durations of approximately 60 and 30 days, respectively. Infection introduction times (*t*_event_) of 90, 160 and 170 days were tested to capture dynamics both prior to and at the onset of extreme aggregation events. When introduced at *t*_event_ = 90, the epidemic is either well established or has died out before aggregation begins. When introduced at *t*_event_ = 160 or *t*_event_ = 170, the infection emerges at the start of an aggregation period. Final size and peak prevalence were recorded for each parameter combination.

**Fig. 4:**
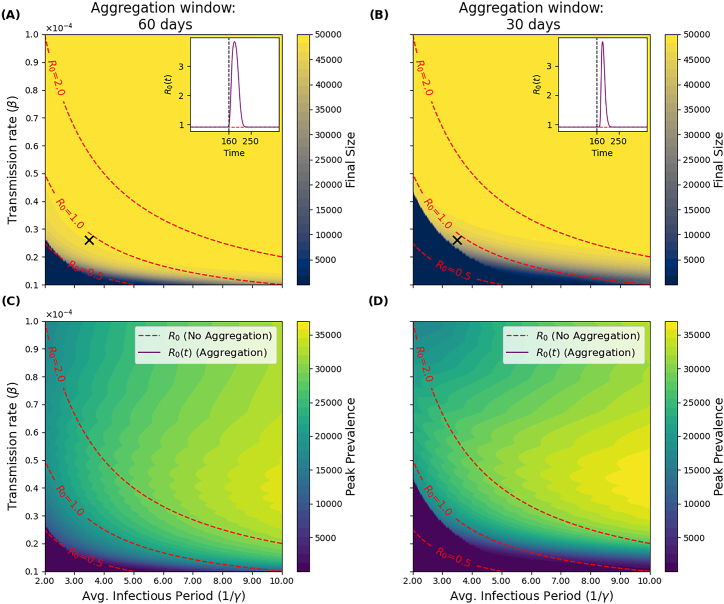
Heatmaps illustrating the final size (Panels A and B) and peak prevalence (Panels C and D) under two aggregation regimes: *b* = 10 and *b* = 20. Infection was introduced at *t*_event_ = 160 days. Red dashed contours represent the baseline basic reproduction number $R_{0}=\beta N/\gamma$ in the absence of aggregation. The black cross marks the parameter pair (*γ* = 1/3.5, *β* = 2.6 × 10^−5^ day^−1^) used for the insets; in each inset, the solid purple curve shows $R_{0}\left(t\right)$ with aggregation, and the red dashed line shows the constant “no aggregation”.$R_{0}$

Fig. 4 presents heatmaps of final size and peak prevalence for *b* = 10 (left column) and *b* = 20 (right column) for an infection introduction at *t*_event_ = 160 days. In Panel A, aggregation creates a sharp shift in final sizes at the contour line $R_{0}=0.5$ (no aggregation), where if an epidemic does occur, almost all of the population are infected. In Panel B, there is a similar effect from aggregation, but it occurs closer to the contour $R_{0}=1$ (no aggregation). Panels C and D show analogous patterns for peak prevalence, where a wider aggregation period leads to a sharp change in peak prevalence at lower $R_{0}$ values. The highest values of peak prevalence occur when the average infectious period is long, but transmission is intermediate. Panels A and B feature a black cross at *γ* = 1/3.5, *β* = 2.6 × 10^−5^, indicating the parameter set used in the $R_{0}\left(t\right)$ insets. These insets compare the time-varying reproduction number under aggregation, $R_{0}\left(t\right)$ (Equation (23), against the constant baseline $R_{0}=\beta \;N/\gamma$ (red dashed line). For the marked parameter pair, shortly after infection introduction at *t*_event_ = 160 days, aggregation coincides with $R_{0}\left(t\right)$ exceeding the critical threshold of 1, peaking near 4. This temporary elevation of $R_{0}\left(t\right)$ above unity explains how density surges enable outbreaks for pathogens with otherwise low baseline $R_{0}$ values.

**Fig. 5:**
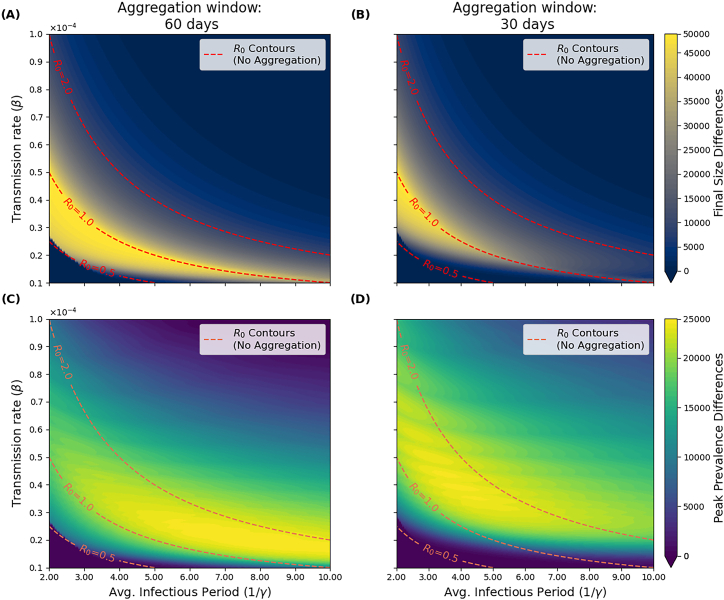
Heatmaps illustrating the differences (Equations (11), (12)) in final size (Panels A and B) and peak prevalence (Panels C and D) under two aggregation regimes: *b* = 10 and *b* = 20, for a closed metapopulation with *N* = 50, 000. Infection was introduced near the onset of aggregation: *t*_event_ = 160 days for *b* = 10 (Panels A,C) and *t*_event_ = 170 days for *b* = 20 (Panels B,D). Red dashed contours represent the baseline $R_{0}=\beta N/\gamma$ (levels 0.5, 1.0, 2.0) in the absence of aggregation. Values near zero indicate little or no difference between Aggregation and No Aggregation; larger values mark regions where aggregation has the strongest effect on the outcome.

Fig. 5 shows the difference in final size and peak prevalence (Equations (11), (12)). The left column corresponds to an aggregation regime of *b* = 10 with infection introduced at *t* = 160 days, while the right column corresponds to *b* = 20 with infection introduced at *t* = 170 days. From Panels A and B it is clear that aggregation has a dramatic impact on final size when $R_{0}$ is between 0.5 and 1. In contrast, the impact of aggregation on peak prevalence tends to be greatest when $R_{0}$ is between 1 and 2. By comparing Panels A and C with B and D, we see that the parameter space where aggregation amplifies epidemics varies with the length of the aggregation and the time of introduction.

**Fig. 6:**
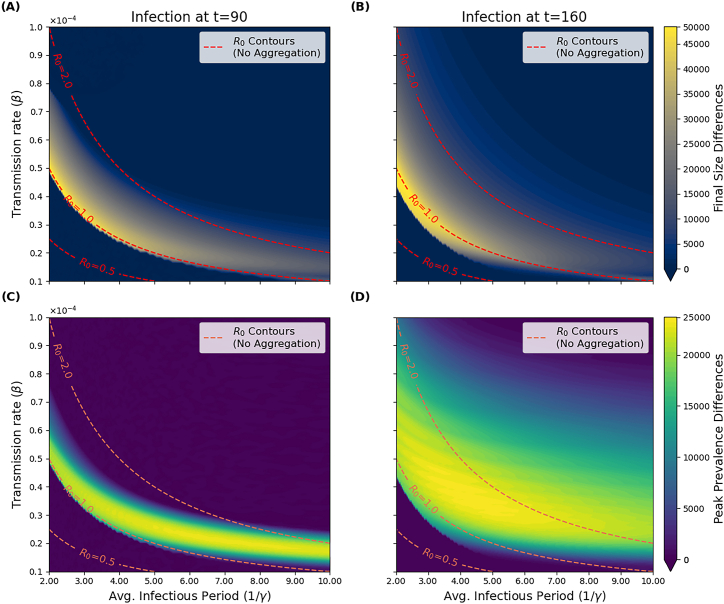
Heatmaps illustrating the differences (Equations (11), (12)) in final size (Panels A and B) and peak prevalence (Panels C and D) for a closed metapopulation with *N* = 50, 000 under an aggregation duration *b* = 20. Infection was introduced at *t*_event_ = 90 days (Panels A,C) and *t*_event_ = 160 days (Panels B,D). Red dashed contours represent the baseline $R_{0}=\beta N/\gamma$ (levels 0.5, 1.0, 2.0) in the absence of aggregation. Values near zero indicate little or no difference between scenarios; larger values mark regions where aggregation has the strongest effect on the outcome.

For a single aggregation length of *b* = 20 (approximately 30 days), we compared outcomes with infection introduced at *t*_event_ = 90 and *t*_event_ = 160 days. Fig. 6 displays heatmaps of the difference in final size (Panels A–B) and peak prevalence (Panels C–D), calculated in a similar manner as Fig. 5. In Panel A (*t*_event_ = 90), aggregation elevates final sizes primarily within the region $1<R_{0}<2$, producing the largest increases just above $R_{0}=1$. When introduction is delayed to *t*_event_ = 160 (Panel B), aggregation has a bigger impact on outbreaks where the baseline $R_{0}<1$, yielding increased final size differences below the $R_{0}=1$ contour. Panels C and D show differing patterns for peak prevalence. At *t*_event_ = 90 (Panel C), peak-prevalence increases are concentrated in the $1<R_{0}<2$ region. For *t*_event_ = 160 (Panel D), the results are similar to that of Panel D in Fig. 5.

## 4 Discussion

This study set out to explore the impact of extreme seasonal aggregation on a non-fatal epidemic in a closed population, to help us better understand outbreak dynamics in wildlife who display these aggregating behaviours. To achieve this we ran an SIR process on a metapopulation with a hub and satellite structure. Seasonal aggregations of varying durations were simulated using a Gaussian function and we also explored the impact of different infection introduction times. For a fixed aggregation period the amplification of both final size and peak prevalence is largest when the pathogen is introduced just before or at the onset of the aggregation (Becker et al., 2020). Infection introductions occurring well before aggregation yield negligible effects, since only pathogens with $R_{0}>1$ persist long enough to benefit from increased mixing while those with $R_{0}<1$ have already died out. However, pathogens that would otherwise fade out after a peak (in the absence of aggregation), can be rescued by the increased influx of individuals (see Fig. 2). Aggregation increases final size when $R_{0}<1$ and increases peak prevalence primarily in the window $1<R_{0}<2$. Finally, the extent of these effects varies with the interaction between the duration of the aggregation pulse and the timing of introduction. Most clearly, extreme increases in local density can rescue or boost outbreaks that would otherwise fade in the absence of aggregation.

Our simulations show the impact of aggregation is largest when pathogen transmissibility is near or slightly below the critical threshold $\left(R_{0}=\beta \;N/\gamma \approx 1\right)$. In these parameter regions, the introduction of aggregation temporarily raises the time dependent basic reproduction number, $R_{0}\left(t\right)$ (see Equation (23)), above this threshold, significantly amplifying the epidemic's magnitude. Consequently, outbreaks that would otherwise fail to take off are transformed into major epidemics. This highlights that the timing of aggregation events are particularly influential in driving severe epidemic outcomes under conditions close to the threshold for sustained transmission.

Our findings concerning the impact of aggregation timing align with observations from complex wildlife disease systems. Modelling of Nipah virus dynamics in *Pteropus* bats has revealed that smaller colonies exhibit $R_{0}$ values around 2.1, whereas larger roosts with higher population densities can drive $R_{0}$ values close to 3.5, emphasising that higher aggregation intensity elevates transmission potential (Epstein et al., 2020). Hendra virus dynamics in Australian flying foxes exhibit pronounced seasonality, with infection pulses and spillover risk often linked to periods coinciding with specific ecological conditions such as episodic shedding, transient epidemics and changes in bat distribution or roosting behaviour (Field et al., 2011; Plowright et al., 2015). While factors beyond simple aggregation density, such as nutritional stress influencing shedding and foraging patterns, are critical drivers of Hendra spillover (Edson et al., 2015; Plowright et al., 2015), our model highlights a complementary principle: if seasonal changes in host distribution (akin to our extreme aggregation events) lead to increased effective contact rates within the flying fox population, the timing of such events relative to the virus's natural transmission cycle within bats could amplify transmission intensity $\left(R_{0}\left(t\right)>1\right)$, contributing to the observed infection pulses. Thus, while our model does not capture the full complexity of Hendra transmission, its results emphasise that understanding the temporal alignment of host aggregation behaviours and pathogen circulation could be a relevant component, alongside other ecological drivers, in explaining seasonal disease patterns.

Similarly, research on migratory waterfowl has demonstrated that dense flocking during breeding and stopover periods leads to significant increases in the transmission of avian influenza viruses (van Dijk et al., 2013; for H5N8 and Viruses, 2016). In these systems, the convergence of numerous birds, many of which are immunologically naïve, produces outbreak surges that align well with the simulation results presented here (Dolfi et al., 2024). Despite the fact that these pathogens generally maintain low prevalence and modest transmission rates, with $R_{0}$ values often just above 1 under natural conditions (Kim & Cho, 2021; Pandit et al., 2013).

The simulation results also find parallels in aquatic systems. Seasonal spawning aggregations of common carp, for example, create well-documented opportunities for pathogens like Cyprinid Herpesvirus 3 (CyHV-3) to spread rapidly, often causing significant outbreaks (Tolo et al., 2023; Uchii et al., 2010). While these field observations establish the general link between aggregation and increased disease, our model provides qualitative insights into how the characteristics of such events influence outcomes. Specifically, the model predicts that the timing of the aggregation relative to the epidemic's progression and the baseline transmissibility $\left(R_{0}\right)$ of the pathogen critically determine the extent to which aggregation amplifies the final size and peak prevalence. For instance, the model suggests that even short, intense spawning events (akin to *b* = 20) could trigger disproportionately large outbreaks if they coincide precisely with the epidemic growth phase, especially for pathogens with lower baseline $R_{0}$ values – a dynamic potentially explaining the explosive nature of some observed CyHV-3 outbreaks.

Despite the encouraging agreement between our model predictions and empirical observations across multiple taxa, several limitations remain. The model assumes a well-mixed (within the metapopulation nodes), closed population with no demography which simplifies the dynamics. For instance, while flying fox colonies are large and dynamic, individuals do move between roosts, and seasonal recruitment of juveniles contributes to a continuously replenished susceptible pool (Vanderduys et al., 2024). Likewise, in migratory birds, movements between different roosts and variable participation in aggregations introduce heterogeneity that is not captured by our homogeneous mixing assumption (Taylor et al., 2011; Vickers et al., 2025). Furthermore, our representation of the aggregation event as a uniform spike in movement rates does not account for differences in individual behaviour or spatial structure within aggregations. In reality, factors such as the centrality of an individual within a group and the local density of contacts will modulate transmission dynamics (Sandel et al., 2020; White et al., 2015). The deterministic nature of our model neglects stochastic effects that may be significant in smaller or more fragmented populations. However, despite these simplifications, the model provides valuable insights into the potential epidemiological consequences of extreme temporary increases in local density. By isolating the effect of aggregation timing and duration under controlled conditions, we clearly demonstrate how these factors can amplify outbreak size and intensity, particularly for pathogens with lower baseline transmissibility $\left(R_{0}<1\right)$, offering a crucial theoretical baseline for understanding and further investigating more complex real-world aggregation phenomena.

## CRediT authorship contribution statement

**Daniel N.R. Longmuir:** Writing – review & editing, Writing – original draft, Visualization, Validation, Software, Methodology, Investigation, Formal analysis, Conceptualization. **Simon Johnstone-Robertson:** Writing – review & editing, Validation, Supervision, Methodology, Conceptualization. **Andrew J. Hoskins:** Writing – review & editing, Resources, Investigation, Conceptualization. **Roslyn I. Hickson:** Writing – review & editing, Visualization, Validation, Methodology, Formal analysis, Conceptualization. **Stephen A. Davis:** Writing – review & editing, Supervision, Project administration, Methodology, Conceptualization.

## Declaration of competing interest

The authors declare that they have no known competing financial interests or personal relationships that could have appeared to influence the work reported in this paper.

## References

[bib1] Becker D.J., Ketterson E.D., Hall R.J. (2020). Reactivation of latent infections with migration shapes population-level disease dynamics. Proceedings of the Royal Society B: Biological Sciences.

[bib2] Berg S.S., Beasley D.E., Bevins S.N., Apley M.D. (2018). Population health in veterinary medicine.

[bib3] Breban R., Drake J.M., Stallknecht D.E., Rohani P. (2009). The role of environmental transmission in recurrent avian influenza epidemics. PLoS Computational Biology.

[bib4] Brown P., Gilligan D. (2014). Optimising an integrated pest-management strategy for a spatially structured population of common carp (cyprinus carpio) using meta-population modelling. Marine and Freshwater Research.

[bib5] Dhirasakdanon T., Sattenspiel L. (2007). A sharp threshold for disease persistence in host metapopulations. Journal of Biological Dynamics.

[bib6] Diekmann O., Heesterbeek J., Metz J. (1990). On the definition and the computation of the basic reproduction ratio r 0 in models for infectious diseases in heterogeneous populations. Journal of Mathematical Biology.

[bib7] Diekmann O., Heesterbeek J.A.P., Roberts M.G. (2010). The construction of next-generation matrices for compartmental epidemic models. Journal of The Royal Society Interface.

[bib8] Dolfi A.C., Kausrud K., Rysava K., Champagne C., Huang Y.-H., Barandongo Z.R., Turner W.C. (2024). Season of death, pathogen persistence and wildlife behaviour alter number of anthrax secondary infections from environmental reservoirs. Proceedings of the Royal Society B: Biological Sciences.

[bib9] Durr P.A., Davis S., Joehnk K., Graham K., Hopf J., Arakala A., McColl K.A., Taylor S., Chen Y., Sengupta A., Merrin L., Stratford D., Aryal S., van Klinken R.D., Brown P., Gilligan D. (2019). Technical report, fisheries research and development corporation, Deakin, Australian Capital Territory. Online resource (vi, 242 pages) : Colour charts, colour maps.

[bib10] Edson D., Field H., McMichael L., Vidgen M., Goldspink L., Broos A., Melville D., Kristoffersen J., de Jong C., McLaughlin A., Davis R., Kung N., Jordan D., Kirkland P., Smith C. (2015). Routes of hendra virus excretion in naturally-infected flying-foxes: I mplications for viral transmission and spillover risk. PLoS One.

[bib11] Epstein J.H., Anthony S.J., Islam A., Kilpatrick A.M., Ali Khan S., Balkey M.D., Ross N., Smith I., Zambrana-Torrelio C., Tao Y., Islam A., Quan P.L., Olival K.J., Khan M.S.U., Gurley E.S., Hossein M.J., Field H.E., Fielder M.D., Briese T., Daszak P. (2020). Nipah virus dynamics in bats and implications for spillover to humans. Proceedings of the National Academy of Sciences.

[bib12] Field H., de Jong C., Melville D., Smith C., Smith I., Broos A., Kung Y.H.N., McLaughlin A., Zeddeman A. (2011). Hendra virus infection dynamics in Australian fruit bats. PLoS One.

[bib13] for H5N8, T. G. C. and Viruses, R. I. (2016). Role for migratory wild birds in the global spread of avian influenza h5n8. Science.

[bib14] Fulford G., Roberts M., Heesterbeek J. (2002). The metapopulation dynamics of an infectious disease: Tuberculosis in possums. Theoretical Population Biology.

[bib15] Grassly N.C., Fraser C. (2006). Seasonal infectious disease epidemiology. Proceedings of the Royal Society B: Biological Sciences.

[bib16] Grear D.A., Hall J.S., Dusek R.J., Ip H.S. (2017). Inferring epidemiologic dynamics from viral evolution: 2014–2015 Eurasian/North American highly pathogenic Avian influenza viruses exceed transmission threshold, r0 = 1, in wild birds and poultry in North America. Evolutionary Applications.

[bib17] Han D., Wei J., Xu H., Li D. (2021). Dynamical analysis of the sis epidemic model in cluster events. Applied Mathematical Modelling.

[bib18] Hanski I. (1999).

[bib19] Hanski I., Gilpin M. (1991). Metapopulation dynamics: Brief history and conceptual domain. Biological Journal of the Linnean Society.

[bib20] Hess G. (1996). Disease in metapopulation models: Implications for conservation. Ecology.

[bib21] Iglesias I., Perez A.M., Sánchez-Vizcaíno J.M., Muñoz M.J., Martínez M., De La Torre A. (2010). Reproductive ratio for the local spread of highly pathogenic avian influenza in wild bird populations of Europe, 2005–2008. Epidemiology and Infection.

[bib22] Jeong J., McCallum H. (2021). The persistence of a sir disease in a metapopulation: Hendra virus epidemics in Australian black flying foxes. Australian Journal of Zoology.

[bib23] Kermack W.O., McKendrick A.G., Walker G.T. (1927). A contribution to the mathematical theory of epidemics. Proceedings of the Royal Society of London - Series A: Containing Papers of a Mathematical and Physical Character.

[bib24] Kermack W.O., McKendrick A.G., Walker G.T. (1932). Contributions to the mathematical theory of epidemics. ii. —the problem of endemicity. Proceedings of the Royal Society of London - Series A: Containing Papers of a Mathematical and Physical Character.

[bib25] Kermack W.O., McKendrick A.G., Walker G.T. (1933). Contributions to the mathematical theory of epidemics. iii.—further studies of the problem of endemicity. Proceedings of the Royal Society of London - Series A: Containing Papers of a Mathematical and Physical Character.

[bib26] Kim W.-H., Cho S. (2021). Estimation of the basic reproduction numbers of the subtypes H5N1, H5N8, and H5N6 during the highly pathogenic avian influenza epidemic spread between farms. Frontiers in Veterinary Science.

[bib27] Levins R. (1969). Some demographic and genetic consequences of environmental heterogeneity for biological control1. Bulletin of the Entomological Society of America.

[bib28] Lloyd A.L., May R.M. (1996). Spatial heterogeneity in epidemic models. Journal of Theoretical Biology.

[bib29] Lloyd-Smith J.O., Cross P.C., Briggs C.J., Daugherty M., Getz W.M., Latto J., Sanchez M.S., Smith A.B., Swei A. (2005). Should we expect population thresholds for wildlife disease?. Trends in Ecology & Evolution.

[bib30] Loiselle B.A., Blake J.G. (1991). Temporal variation in birds and fruits along an elevational gradient in Costa Rica. Ecology.

[bib31] Lunn T.J., Peel A.J., McCallum H., Eby P., Kessler M.K., Plowright R.K., Restif O. (2021). Spatial dynamics of pathogen transmission in communally roosting speci es: Impacts of changing habitats on bat-virus dynamics. Journal of Animal Ecology.

[bib32] McCallum H., Barlow N., Hone J. (2001). How should pathogen transmission be modelled?. Trends in Ecology & Evolution.

[bib33] Olsen B., Munster V.J., Wallensten A., Waldenström J., Osterhaus A.D.M.E., Fouchier R.A.M. (2006). Global patterns of influenza a virus in wild birds. Science.

[bib34] Pandit P.S., Bunn D.A., Pande S.A., Aly S.S. (2013). Modeling highly pathogenic avian influenza transmission in wild birds and poultry in West Bengal, India. Scientific Reports.

[bib35] Peel A.J., Pulliam J.R.C., Luis A.D., Plowright R.K., O'Shea T.J., Hayman D.T.S., Wood J.L.N., Webb C.T., Restif O. (2014). The effect of seasonal birth pulses on pathogen persistence in wild mammal populations. Proceedings of the Royal Society B: Biological Sciences.

[bib36] Phipps W.L., López-López P., Buechley E.R., Oppel S., Álvarez E., Arkumarev V., Bekmansurov R., Berger-Tal O., Bermejo A., Bounas A., Alanís I.C., de la Puente J., Dobrev V., Duriez O., Efrat R., Fréchet G., García J., Galán M., García-Ripollés C., Vallverdú N. (2019). Spatial and temporal variability in migration of a soaring raptor across three continents. Frontiers in Ecology and Evolution.

[bib37] Plowright R.K., Eby P., Hudson P.J., Smith I.L., Westcott D., Bryden W.L., Middleton D., Reid P.A., McFarlane R.A., Martin G., Tabor G.M., Skerratt L.F., Anderson D.L., Crameri G., Quammen D., Jordan D., Freeman P., Wang L.-F., Epstein J.H., McCallum H. (2015). Ecological dynamics of emerging bat virus spillover. Proceedings of the Royal Society B: Biological Sciences.

[bib38] Plowright R.K., Foley P., Field H.E., Dobson A.P., Foley J.E., Eby P., Daszak P. (2011). Urban habituation, ecological connectivity and epidemic dampening: The emergence of hendra virus from flying foxes (*pteropus* spp.). Proceedings of the Royal Society B: Biological Sciences.

[bib39] Roberts B.J., Catterall C.P., Eby P., Kanowski J. (2012). Long-distance and frequent movements of the flying-fox pteropus poliocephalus: Implications for management. PLoS One.

[bib40] Roche B., Lebarbenchon C., Gauthier-Clerc M., Chang C.-M., Thomas F., Renaud F., van der Werf S., Guégan J.-F. (2009). Water-borne transmission drives avian influenza dynamics in wild birds: The case of the 2005–2006 epidemics in the camargue area. Infection, Genetics and Evolution.

[bib41] Rushing C.S., Hostetler J.A., Sillett T.S., Marra P.P., Rotenberg J.A., Ryder T.B. (2017). Spatial and temporal drivers of avian population dynamics across the annual cycle. Ecology.

[bib42] Saenz R.A., Essen S.C., Brookes S.M., Iqbal M., Wood J.L.N., Grenfell B.T., McCauley J.W., Brown I.H., Gog J.R. (2012). Quantifying transmission of highly pathogenic and low pathogenicity h7n1 avian influenza in turkeys. PLoS One.

[bib43] Sandel A.A., Rushmore J., Negrey J.D., Mitani J.C., Lyons D.M., Caillaud D. (2020). Social network predicts exposure to respiratory infection in a wild chimpanzee group. EcoHealth.

[bib44] Smith M.J., Telfer S., Kallio E.R., Burthe S., Cook A.R., Lambin X., Begon M. (2009). Host–pathogen time series data in wildlife support a transmission function between density and frequency dependence. Proceedings of the National Academy of Sciences.

[bib45] Taylor P.D., Mackenzie S.A., Thurber B.G., Calvert A.M., Mills A.M., McGuire L.P., Guglielmo C.G. (2011). Landscape movements of migratory birds and bats reveal an expanded scale of stopover. PLoS One.

[bib46] Tolo I.E., Bajer P.G., Mor S.K., Phelps N.B.D. (2023). Disease ecology and host range of cyprinid herpesvirus 3 (<scp>cyhv</scp>-3) in <scp>cyhv</scp>-3 endemic lakes of North America. Journal of Fish Diseases.

[bib47] Tolo I.E., Bajer P.G., Wolf T.M., Mor S.K., Phelps N.B.D. (2021). Investigation of cyprinid herpesvirus 3 (cyhv-3) disease periods and factors influencing cyhv-3 transmission in a low stocking density infection trial. Animals.

[bib48] Uchii K., Telschow A., Minamoto T., Yamanaka H., Honjo M.N., Matsui K., Kawabata Z. (2010). Transmission dynamics of an emerging infectious disease in wildlife through host reproductive cycles. The ISME Journal.

[bib49] van Dijk J.G.B., Hoye B.J., Verhagen J.H., Nolet B.A., Fouchier R.A.M., Klaassen M. (2013). Juveniles and migrants as drivers for seasonal epizootics of avian influenza virus. Journal of Animal Ecology.

[bib50] Van Rossum G., Drake F.L. (2009).

[bib51] Vanderduys E.P., Caley P., McKeown A., Martin J.M., Pavey C., Westcott D. (2024). Population trends in the vulnerable grey-headed flying-fox, pteropus poliocephalus; results from a long-term, range-wide study. PLoS One.

[bib52] Vickers S.H., Meehan T.D., Michel N.L., Franco A.M.A., Gilroy J.J. (2025). North American avian species that migrate in flocks show greater long-term non-breeding range shift rates. Movement Ecology.

[bib53] Virtanen P., Gommers R., Oliphant T.E., Haberland M., Reddy T., Cournapeau D., Burovski E., Peterson P., Weckesser W., Bright J., van der Walt S.J., Brett M., Wilson J., Millman K.J., Mayorov N., Nelson A.R.J., Jones E., Kern R., Larson E., van Mulbregt P., SciPy 1.0 Contributors (2020). SciPy 1.0: Fundamental algorithms for scientific computing in python. Nature Methods.

[bib54] Wang H.-H., Kung N.Y., Grant W.E., Scanlan J.C., Field H.E. (2013). Recrudescent infection supports hendra virus persistence in Australian flying-fox populations. PLoS One.

[bib55] Welbergen J.A., Meade J., Field H.E., Edson D., McMichael L., Shoo L.P., Praszczalek J., Smith C., Martin J.M. (2020). Extreme mobility of the world's largest flying mammals creates key challenges for management and conservation. BMC Biology.

[bib56] Westcott D.A., McKeown A., Bradford M., Vanderduys E., Hoskins A., Macdonald S.L., Eyre T., Bracks J., Bell K., Hogan L.D., Smith G.C., McVicar T.R., Venz M.F., Pegg G., Thomas K., Shaw P., Brumby M., Abbott B., Batchelor K., Li L. (2020).

[bib57] White L.A., Forester J.D., Craft M.E. (2015). Using contact networks to explore mechanisms of parasite transmission in wildlife. Biological Reviews.

